# Simple Linear Cancer Risk Prediction Models With Novel Features Outperform Complex Approaches

**DOI:** 10.1200/CCI.21.00166

**Published:** 2022-03-03

**Authors:** Scott Kulm, Lior Kofman, Jason Mezey, Olivier Elemento

**Affiliations:** ^1^ Caryl and Israel Englander Institute of Precision Medicine, Weill Cornell Medicine, New York, NY; ^2^ Physiology, Biophysics and Systems Biology Graduate Program, Weill Cornell Medicine, New York, NY; ^3^ Department of Computer Science, Tufts University, Medford, MA; ^4^ Department of Genetic Medicine, Weill Cornell Medicine, New York, NY; ^5^ Department of Computational Biology, Cornell University, Ithaca, NY

## Abstract

The ability to accurately predict an individual's risk for cancer is critical to the implementation of precision prevention measures. Current cancer risk predictions are frequently made with simple models that use a few proven risk factors, such as the Gail model for breast cancer, which are easy to interpret, but may theoretically be less accurate than advanced machine learning (ML) models.

**METHODS:**

With the UK Biobank, a large prospective study, we developed models that predicted 13 cancer diagnoses within a 10-year time span. ML and linear models fit with all features, linear models fit with 10 features, and externally developed QCancer models, which are available to more than 4,000 general practices, were assessed.

**RESULTS:**

The average area under the receiver operator curve (AUC) of the linear models (0.722, SE = 0.015) was greater than the average AUC of the ML models (0.720, SE = 0.016) when all 931 features were used. Linear models with only 10 features generated an average AUC of 0.706 (SE 0.015), which was comparable to the complex models using all features and greater than the average AUC of the QCancer models (0.684, SE 0.021). The high performance of the 10-feature linear model may be caused by the consideration of often omitted feature types, including census records and genetic information.

**CONCLUSION:**

The high performance of the 10-feature linear models indicate that unbiased selection of diverse features, not ML models, may lead to impressively accurate predictions, possibly enabling personalized screening schedules that increase cancer survival.

## INTRODUCTION

Accurate estimation of an individual's risk for cancer is a prerequisite to change the current, largely one-size-fits-all method of care to a far more personalized approach with improved health outcomes.^1^ This current paradigm of care schedules cancer screening largely according to an individual's age, an overly simplistic approach.^2-5^ Several more advanced linear models have been introduced that combine multiple known risk factors, including the National Cancer Institute's Risk Assessment Tools,^6,7^ which includes the Gail model, and the University of Oxford's QCancer models.^8^ Although these linear models are more accurate than an age-only approach, improvements in both data availability and computational power suggest that more sophisticated models may perform better. One clear attempt to prove this point is the application of machine learning (ML) algorithms, which can be trained either directly on the basis of data from an existing electronic health record, or from data available in large biobanks that are coupled with extensive phenotype data.^9-11^ ML models using biobank data are advantageous, because individuals are followed for many years and these biobanks can be quite large, as exemplified by the UK Biobank, with more than 500,000 participants^9^ and the upcoming All of Us, with up to a 1 million diverse participants.^12^ The largest disadvantage of an ML approach for predicting risk is the limited interpretability of ML models. Although linear models are sometimes considered less accurate than ML approaches, they are more easily interpretable than ML-based approaches. To the best of our knowledge, they have not yet been fully developed with the full breadth of biobank data and compared with ML and existing cancer risk prediction approaches. Current comparisons between linear and ML models^13^ lead us to hypothesize that linear models trained on a small set of the most salient features can rival or exceed the accuracy of ML and other currently available models.

**CONTEXT**

**Key Objective**
Current methods for cancer risk prediction often use a few, manually selected features. To test whether improved methodologies exist, we fit multiple model types for 13 cancers upon the UK Biobank, a 500,000-person cohort with diverse data.
**Knowledge Generated**
Linear models with 10 features were nearly as predictive as both advanced machine learning models and linear models with more than 100 features, while being significantly more predictive than the traditionally derived QCancer models.
**Relevance**
Clinicians planning to generate an interpretable cancer risk model should consider applying unbiased computational algorithms upon a large diverse data set, resulting in an accurate, linear model with a modest number of features.

## METHODS

**FIG 1.**
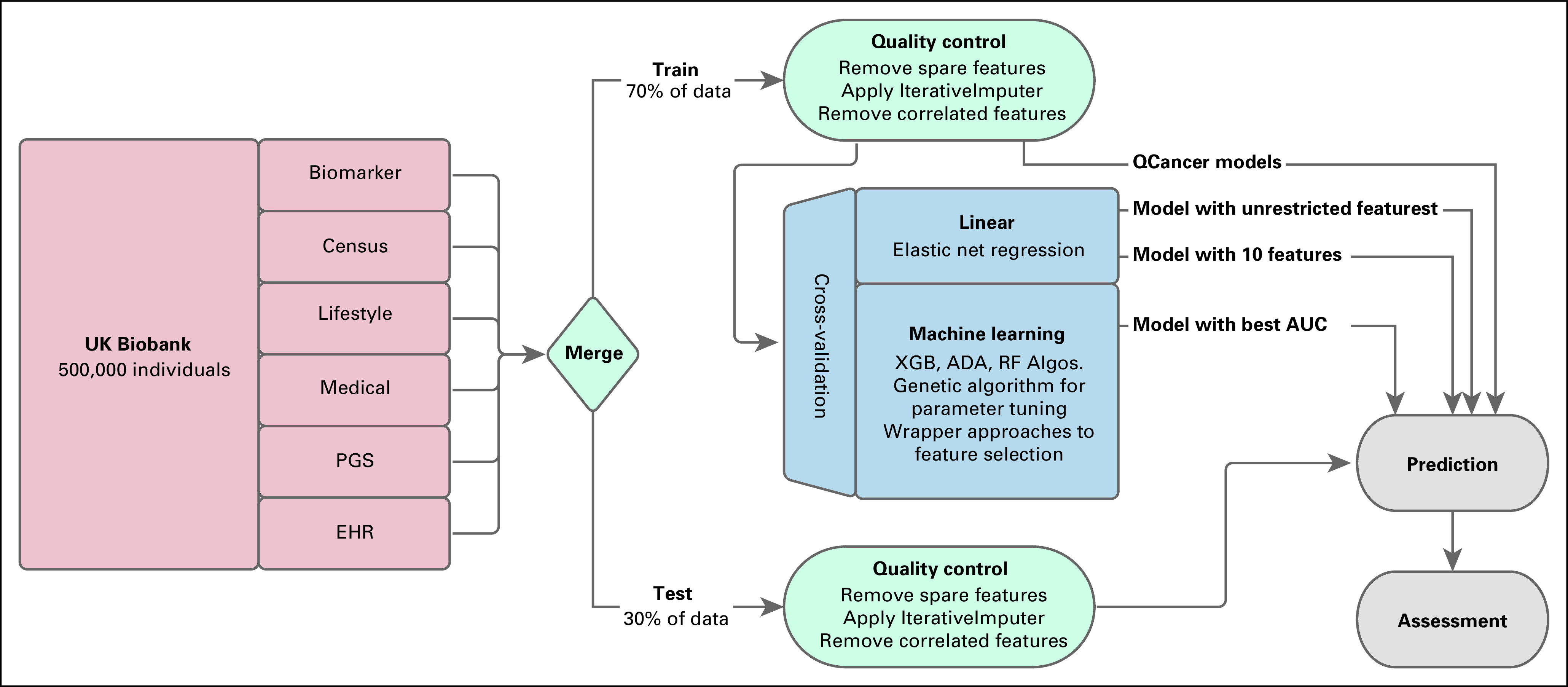
Flow chart of the process that derived the cancer risk prediction models and the following assessment. Additional methodologic details are recorded in the Data Supplement, including figures. ADA, AdaBoost; Algos, algorithms; AUC, area under the receiver operator curve; EHR, electronic health record; PGS, polygenic score; RF, random forest; XGB, XGBoost.

The UK Biobank,^9^ a prospective cohort of more than 500,000 individuals age 40-69 years when assessed between 2006 and 2010, provides all necessary data to train and evaluate our hypothesis (Fig 1). We began model creation by manually extracting six varieties of data, totaling 931 features: medical, lifestyle, electronic health records, biomarkers, census data, and cancer polygenic risk scores (PGSs).^14^ All the features were recorded on or before the day of UK Biobank assessment. Any individual with a diagnosis indicating previous cancer was removed (Data Supplement).

Twelve cancer outcomes were established using the SEER definitions^15^ (Data Supplement). These cancers were chosen because they all have available PGSs^14^ and SEER-defined conversions from International Classification of Diseases-10 codes to cancer labels. Additionally, all leukemia was included to provide a valuable comparison to lymphocytic leukemia and non-Hodgkin lymphoma. The 10-year time frame began for each individual on their date of assessment. Individuals were filtered to those with White-British ancestry to maximize PGS efficacy. Next, features were removed that either had more than 75% of values missing or had a correlation above 0.95 to another feature. Then, the data underwent a 70:30 split between training and testing sets. The number of cases and controls in the testing set is indicated in Table 1, with descriptive statistics provided in the Data Supplement.

**TABLE 1.**
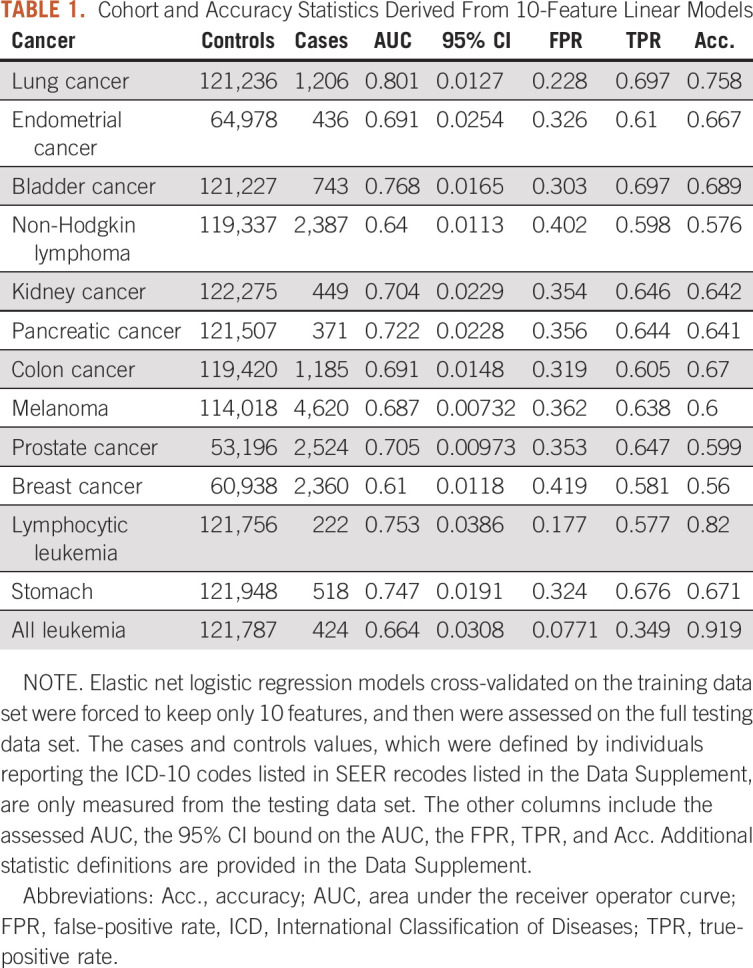
Cohort and Accuracy Statistics Derived From 10-Feature Linear Models

Random forest^16^ (RF), AdaBoost^16^ (ADA), XGBoost^17^ (XGB), and elastic net logistic regression^16^ models were used to predict cancer outcomes from the cleaned feature set. Starting with all 931 available features, a genetic algorithm fit multiple models, each with their own set of hyperparameters. Accuracy was determined through cross-validation within the training data set. A subset of the most important features in the best performing model became the new feature set, and the process was repeated. In this manner, we assessed various subsets of features simultaneously, in an unbiased, algorithmic manner. The model, features, and parameters that produced the highest area under the receiver operator characteristic curve (AUC) was fit to the total training set and assessed on the testing set.

ElasticNet logistic regression models were similarly developed through cross-validation within the training data set. This embedded approach fits a logistic regression model upon the training fold, iteratively attempting to optimize the feature coefficients while being penalized for the number of nonzero coefficients. This penalization effectively selects a subset of feature for inclusion in the model. An AUC is computed corresponding to the fit model. The cross-validation folds are then alternated, and the model is refit and re-evaluated. This entire process then repeats with a different level of penalization. The penalization corresponding to the greatest average AUC is used to fit an ElasticNet model across the entire training data set, thereby generating the full linear model. The 10-feature linear model is generated by selecting the level of penalization that leaves only 10 nonzero feature coefficients.

Although ML models could be limited by the genetic algorithm to 10 features in the process of feature selection, they were not, unless their cross-validation AUC was highest. Similarly, although ElasticNet models with five or 15 features could have been fit, we only evaluated those with 10. Both steps were taken for the same two reasons. First, for each cancer we wanted to assess a single, small-feature-set, linear model that would align with those currently in clinical use, such as the QCancer models, whose median number of features was 10. Second, because we did not want to assess the testing data set more than necessary, as doing otherwise would lead to overfitting and improper accuracy estimates.

Prefit linear models provided from QCancer^18^ were assessed on the testing data set by applying all necessary features (Data Supplement).

Postprediction assessments of model accuracy generated receiver operator characteristic curves (ROCs) by applying the pROC library and odds ratios by partitioning individuals into exposed and nonexposed groups on the basis of specified risk cutoffs. The features within the models were assessed by their model-derived importance, XGB-derived gain, and their linear coefficients. More detailed methods are described in the Data Supplement, including a key for all used acronyms (Data Supplement), and additional data are located in the online data section.

All UK Biobank participants provided full written informed consent for data collection and analysis at study recruitment. This study had ethics approval as part of overall UK Biobank ethics approval (NHS National Research Ethics Service). We undertook the study under UK Biobank Access Application 47137.

## RESULTS

**FIG 2.**
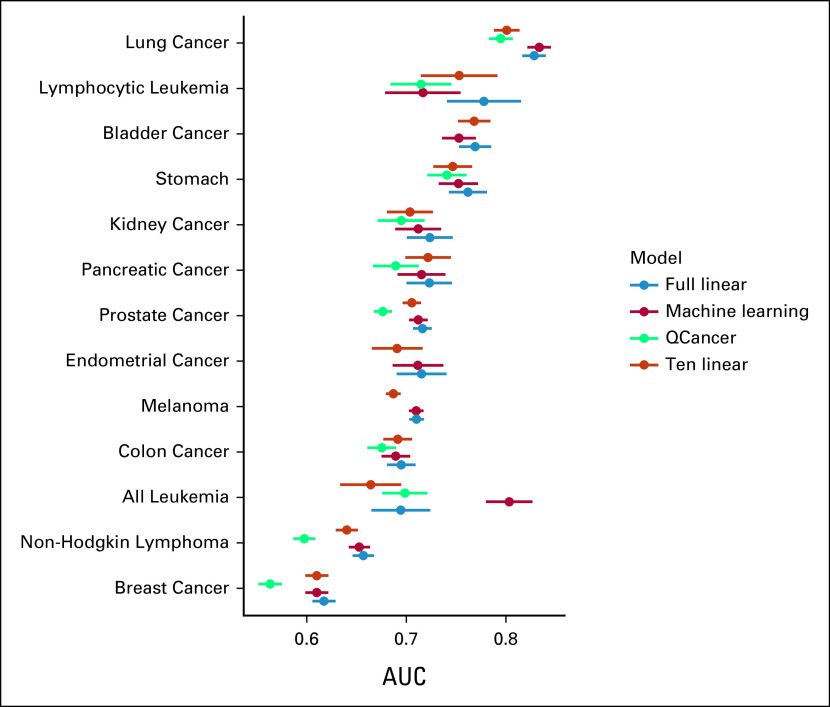
AUC values for each cancer and model assessed in the withheld testing data set. The error bars indicate 95% CI. Several noteworthy patterns in model performance are contained in this figure. The full linear model performs insignificantly different from the machine learning model, the performance difference between 10-feature and full linear model is small enough to suggest that models with more than 10 features are not necessarily justified, and the QCancer traditional model performs significantly worse than all other models. AUC, area under the receiver operator curve.

To assess the accuracy of multiple cancer risk prediction models, we leveraged the UK Biobank. Risk models were built against each of 13 respective cancer diagnoses recorded after the assessment data. We first sought to determine the performance of the externally derived and clinically used QCancer models. Each QCancer model requires between 7 and 16 features (Data Supplement), which may include age, smoking status, diagnosis of Type I diabetes, and Townsend deprivation index. The average AUC of the QCancer models was 0.684 (SE 0.021; Fig 2, Data Supplement).

We then sought to build ML models from the same data set. For each of the 13 cancers, cross-validation within 70% of the data set selected the ML model with the highest area under the receiver operating characteristic curve (AUC) among the algorithms of XGB, AdaBoost, and random forest. The XGB algorithm was selected most often, in eight cancers, followed by AdaBoost in four cancers, and lastly, random forest in a single cancer, lymphocytic leukemia (Data Supplement). When these ML models were applied to the withheld 30% of the data set they generated an average AUC of 0.720 (SE 0.016). Breast cancer had the lowest AUC of 0.610 (95% CI, 0.599 to 0.622) and lung cancer had the highest AUC of 0.833 (95% CI, 0.822 to 0.845). The predictive accuracy of a specific cancer can be partially explained by its sample size and etiologic complexity; for example, lung cancer is easily predicted due in part to the high impact of smoking. However, various poorly understood factors prevent a complete explanation for why any single cancer is more or less easily predicted than another cancer.

Along with ML models, a more simplistic logistic regression model was similarly fit to the full data set and assessed for all cancers, generating an average AUC of 0.722 (SE 0.015). Thus, on average, logistic regression slightly outperformed the ML models. Specifically, the average difference between each cancer's linear and ML AUC was 0.0013 (SE 0.0102). Only lung cancer's and all leukemia's ML model outperformed its linear model by a difference of 0.005 and 0.108, respectively (Fig 2, Data Supplement). The exceptionally large difference for all leukemia may be motivated by its relatively large sample size and complex etiology that can be well represented only through ML models. When comparing predictions via ROCs, two linear models were significantly better than their respective ML models, 10 linear models were insignificantly different than their respective ML models, and one linear model was significantly worse than its respective ML model (Data Supplement). Therefore, linear models may not always be tremendously better than ML models, but they are on average the preferred approach.

On average, 148.5 (SE 15.3) features were included in the full linear models, a number that may be too large to reasonably collect in a clinical setting (Data Supplement). To determine whether a more clinically practical linear model with fewer features could compete with ML predictions, we fit a logistic regression model for each cancer with only 10 features. We chose 10 because it was in the range of the number of features included in the clinically established QCancer models and allowed for consistent simple model comparisons across cancers. The 10-feature linear models generated an average AUC of 0.706 (SE 0.015; Table 1). The average difference between the 10-feature linear model's AUC and the full data set ML model's AUC was –0.014 (SE 0.011) and the average difference between the 10-feature linear model's AUC and the full data set linear model's AUC was –0.016 (SE = 0.003; Fig 2, Data Supplement). When comparing 10-feature linear model predictions to ML predictions via ROCs, six cancers were predicted significantly better by ML models, one cancer was predicted significantly better by 10-feature linear models, and six cancers were predicted insignificantly different (Data Supplement). The wide variation in model superiority can be partially explained by some cancers having relative few, highly penetrant risk factors, whereas other cancers have a wide array of risk factors with modest effect, each of which may interact with each other. Although the ML models are on average better than the 10-feature linear models, the relatively small and imprecise average difference in accuracy likely does not compensate for their long training time and limited interpretability.

The average difference between the 10-feature linear model's AUC and the QCancer model's AUC was 0.019 (SE 0.008; Fig 2, Data Supplement). The definite improvement of the 10-feature linear model over the QCancer model indicates that a diverse set of unbiased features can lead to better predictions than a small, curated set of features. Furthermore, the fact that QCancer models are used clinically indicates that the methodology behind the 10-feature linear, full linear, and ML models may also be clinically relevant.

Although the full linear models are superior according to the AUC statistic (Fig 2, Data Supplement), other statistics, such as odds ratios, may favor a different class of models. Odds ratios were specifically calculated in a contingency table where the exposed individuals had predictions above the specified quantile and nonexposed individuals had predictions below the 50th percentile. When specifying exposed individuals as having a prediction above the 95th percentile, the average odds ratio for QCancer was 4.75 (SE 1.28), for ML models was 10.65 (SE 1.22), for full linear models was 10.78 (SE 1.20), and for 10-feature linear models was 8.84 (SE 1.18). The odds ratio of the ML model was greater than the odds ratio of the full linear model for five cancers and greater than the odds ratio of the 10-feature linear model for 12 cancers (Data Supplement). Although odds ratios well represent the risk contained in a strata, they poorly account for the prevalence of disease. We therefore formed precision-recall curves, which better represent a model's accuracy with respect to the total population under assessment (Data Supplement). All accuracy metrics, odds ratios, precision-recall curves, and ROC curves align on the question of model-type superiority. Full linear models are the most accurate among all models analyzed, and the 10-feature linear models are comparable as a Wilcoxon test found no significant difference between samples of odds ratios generated by each type of model.

The 10-feature linear models contain features that are well understood as well as features that have never been used before. To get a better understanding of which type of features most influenced the model prediction, six categories were established.

**FIG 3.**
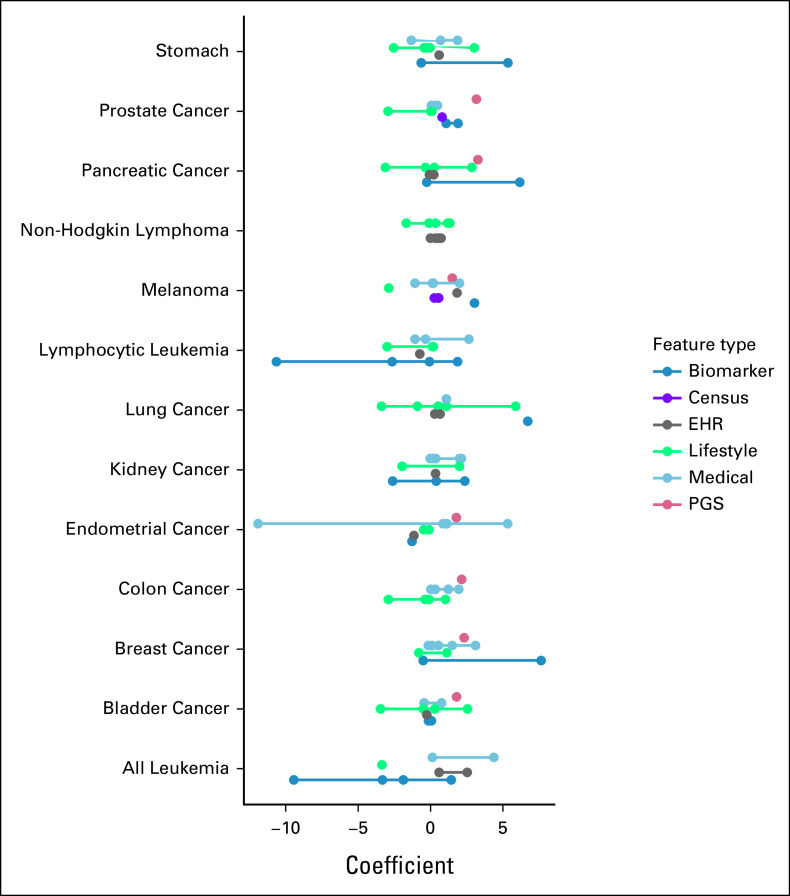
Feature importance to predicting cancers. For each 10-feature linear model, the feature's model coefficients are displayed, colored by the category of the feature. The horizontal bars delineate which features correspond to which cancers, and the colored lines visually group together the class of feature for a given cancer.

**FIG 4.**
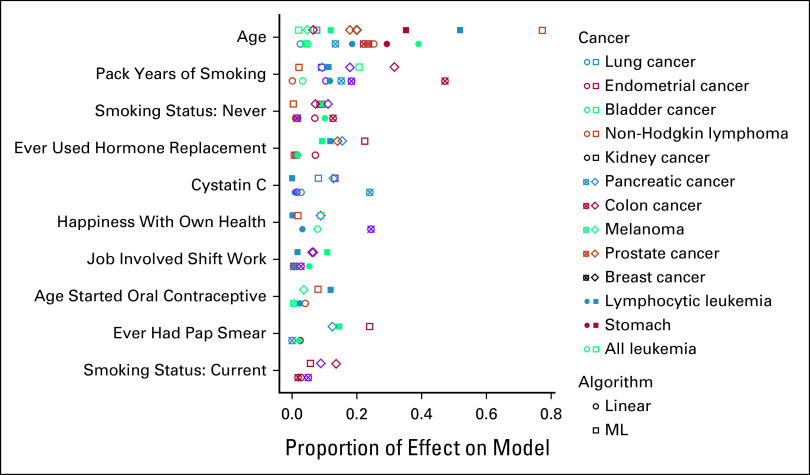
Comparison of features across linear and ML models. The proportion of effect on model is calculated as either the absolute value of the feature's coefficient in the 10-feature linear model divided by the sum of absolute coefficients of the 10-feature linear model, or the feature's importance in the ML model divided by the sum of importances of the ML model. All square points represent the ML model and the circle points represent the 10-feature linear model. As some features were not used for all cancers, fewer than 13 circle and 13 square points are shown for each row. The 10 most common features are displayed. The effects of all features are listed in the Data Supplement. ML, machine learning.

The biomarker category, which includes cholesterol and white blood cell count, contributed 59.9% of the total sum of absolute value coefficients across all cancers. Medical features originated 15.3% of the sum and lifestyle features originated 16.5% of the sum (Fig 3). The three most common features include age, sex, and pack-years of smoking—all common risk features. However, novel features such as cystatin c and happiness with own health were also common, included in five and three models, respectively (Fig 4, Data Supplement). The ability of these features to empower accurate 10-feature linear models suggests that they should be more closely examined and considered in future analyses.

ML models may identify different salient features compared with linear models because their tree-based structure provides a nonlinear approach to model fitting. However, we found that many of the dominant features in the linear models re-emerged in the ML models. For example, age and smoking were the most common ML features, but age was weighted far more heavily in ML models compared with 10-feature linear models. Of the sum of ML absolute importance values across all cancers, age contributed 19.6% to the total, whereas of the sum of 10-feature linear absolute coefficients across all cancers, age contributed only 15.9% to the total. Other interesting and novel features also surfaced, such as census count of individuals whose highest education is apprenticeship and the count of cars in the household (Fig 4, Data Supplement), potentially providing valuable leads in future investigations. Unlike linear models, some ML models can efficiently detect feature interactions, an often unconsidered type of potential risk factor. Specifically, interactions from the XGB model were tested within a linear model. From 833 interactions available across 13 cancers, 46 were significant after Bonferroni correction (Data Supplement). Despite the significance of the interactions, their inclusion in the 10-feature linear model only marginally increased its accuracy. The average difference between the AUC of the 10-feature linear model with the 10 most significant interactions and the same model without interactions is –0.0072 (SE 0.014). This minor decrease in accuracy exemplifies how beneficially introducing interactions into a model is difficult. The 10-feature linear model should therefore serve as an ideal approach for future, clinical-facing cancer investigations.

## DISCUSSION

This analysis of cancer risk within the UK Biobank shows that a simple linear model of diverse features is superior to both advanced ML and traditional approaches. For a majority of cancers, the 10-feature linear models were both significantly more accurate than the traditional QCancer models and insignificantly more accurate than the complex ML models. As these models have not been rigorously validated in multiple independents cohorts, they are not intended for direct clinical application, but rather are meant to illustrate the efficacy of the model derivation procedures. Therefore, a clinician seeking a model for cancer prediction would find the 10-feature linear model methodology we have used to likely be an optimal choice, as it provides greater performance than current approaches while not requiring any complicated algorithm.

The strong predictive capabilities of linear models built from a large, diverse data set have been hinted at, although never fully examined, within other studies. For example, an investigation that used the same cancers as our own found that linear models containing a wide range of features, including age, family history, and lifestyle factors, saw accuracy improvements by increasing data diversity through the addition of PGSs.^19^ Another investigation started with a large, diverse data set from the UK Biobank and, through the application of modeling methodologies similar to our own, was able to predict cardiovascular disease significantly better than the Framingham risk score, generating a C-statistic of 0.796.^20^ However, most investigations do not develop models through computational feature selection algorithms applied to large data sets, rather they simply handpick features thought to be important.^7,21,22^ Although this approach was previously necessary because of computational and resource limitations, and has generated useful results, it should likely no longer be considered the preferred way to create clinical models because it is less accurate than approaches that unbiasedly select features from a diverse data set.

The largely unbiased, big-data–based model development techniques that we recommend may select features that are often excluded from other, traditional cancer prediction models, such as self-reported happiness with own health or waist circumference. These types of features do have strong statistical evidence for their inclusion in the model, as their removal would cause a drop in prediction accuracy. Although most features also have well-cited, biologic links to cancer, such as smoking to lung cancer, a few features do not, as they instead describe an individual profile that is on average significantly more or less likely to be diagnosed with cancer. For example, an individual who undergoes a pap smear is not linked to increased lung cancer risk through an obvious, describable mechanism, but rather might be more likely to be in an environment where lung cancer can be diagnosed because of their apparent participation in preventative health care, a more roundabout connection to cancer risk. However, the utility of these indirect features may be limited to the population they are discovered in, and should be carefully examined and used to ensure the model performs equitably across all groups.

The general applicability of our results does face certain limitations. First, we only analyzed a single biobank, and a single ancestry, making it unclear whether our findings could apply to other groups. However, care was taken to prevent overfitting through cross-validation and only statistically well-powered cancers were examined. Second, our general recommendation to develop simple models from a diverse data set may not generate the best predictions for all cancers. When a large sample size is available or a complex disease etiology is present, then ML models may perform best. Third, certain health care systems may not currently have the data-collection capabilities to enable anything more than a simple, traditional approach. In this instance, we recommend the features we found to have a relatively high effect on the model and strong supporting evidence be prioritized for future data collection and evaluation.

Although simple, linear cancer risk prediction models derived from large, diverse data sets are not necessarily easy to develop because of the initial labor needed to record and cleanse the extensive number of features, they are more interpretable than complex ML approaches and, our results would suggest, more accurate than traditional approaches. We therefore believe that investigators who seek the best-possible, clinically oriented cancer model should first consider our simple, linear methodology. Future related work would involve validation studies that form the model types we recommend with real-world data sources, such as a hospital's electronic health records or a general practitioner's medical charts, and then compare the resultant predictions against the current, local approach taken to designate cancer risk. If the model predictions are more accurate than the current approaches, our recommendation could possibly lead to improved clinical care.
